# Supplementary figures and images for: Adaptations of an RNA virus to increasing thermal stress
Source: PLoS One. 2017 Dec 21;12(12):e0189602. doi: 10.1371/journal.pone.0189602 (PMC5739421; doi:10.1371/journal.pone.0189602)

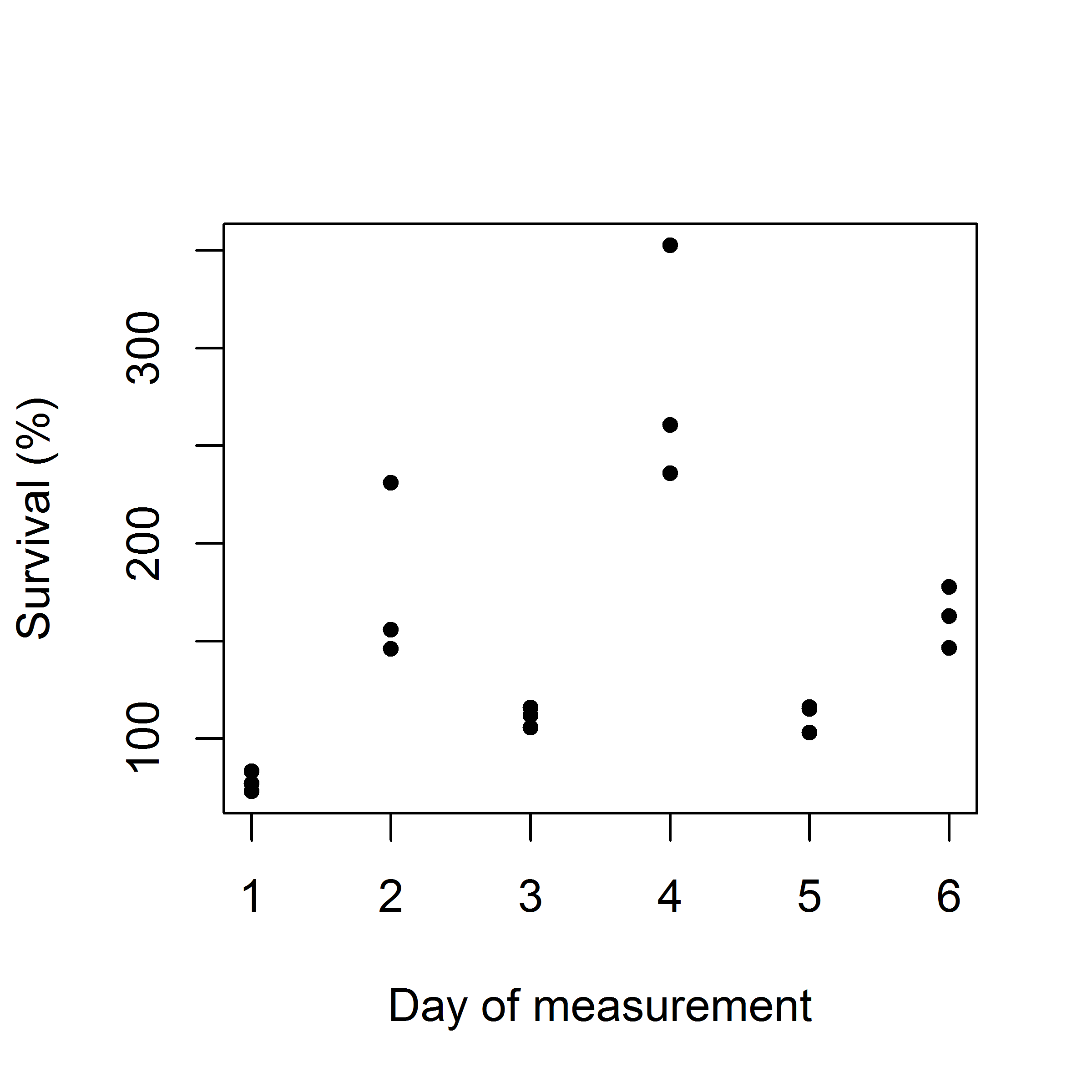

Supplement: S1 Fig — The survival of three replicates was measured on six different days. On five of the days, measured baseline survival of the ancestor at its typical growth temperature was greater than 100%. (TIF) [file pone.0189602.s005.tif]

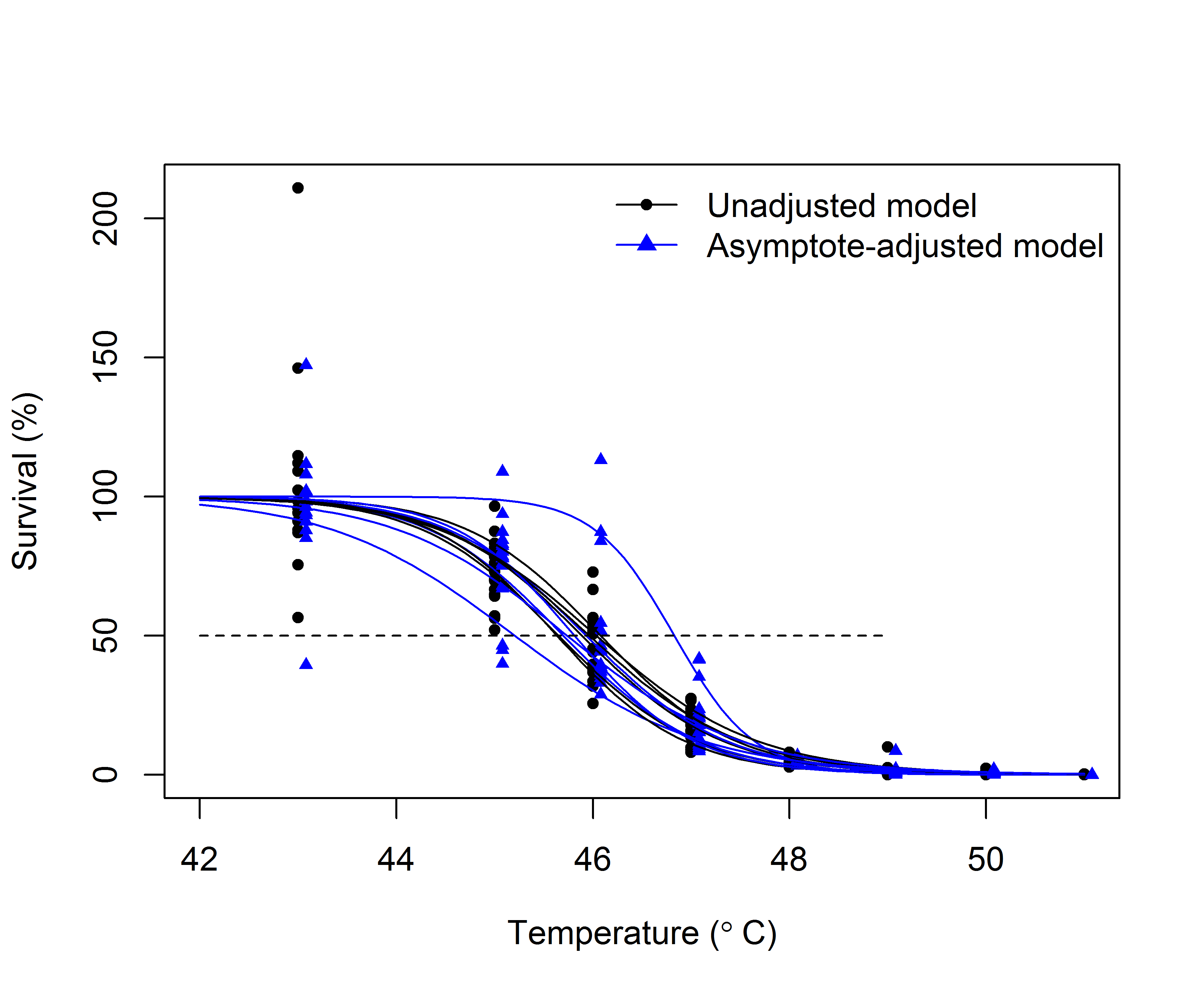

Supplement: S2 Fig — Temperatures from 42–51°C are shown to highlight the region where survival decreased the most. Survivals were taken across the range of heat shock temperatures on six different days. Eq 1 was fit to each day separately. The unadjusted model (black lines) used the survival data as measured (black circles), fixing the asymptote (parameter a) at 100%. For the asymptote-adjusted model, parameter a was allowed to vary across days. Survival values were then divided by their respective asymptote (blue triangles; offset horizontally for better visualization), and Eq 1 re-fit to the adjusted data (blue lines). T50 values (intersection of the curves with the dotted line) were more similar across days when the data were not adjusted for varying asymptotes. (TIF) [file pone.0189602.s006.tif]

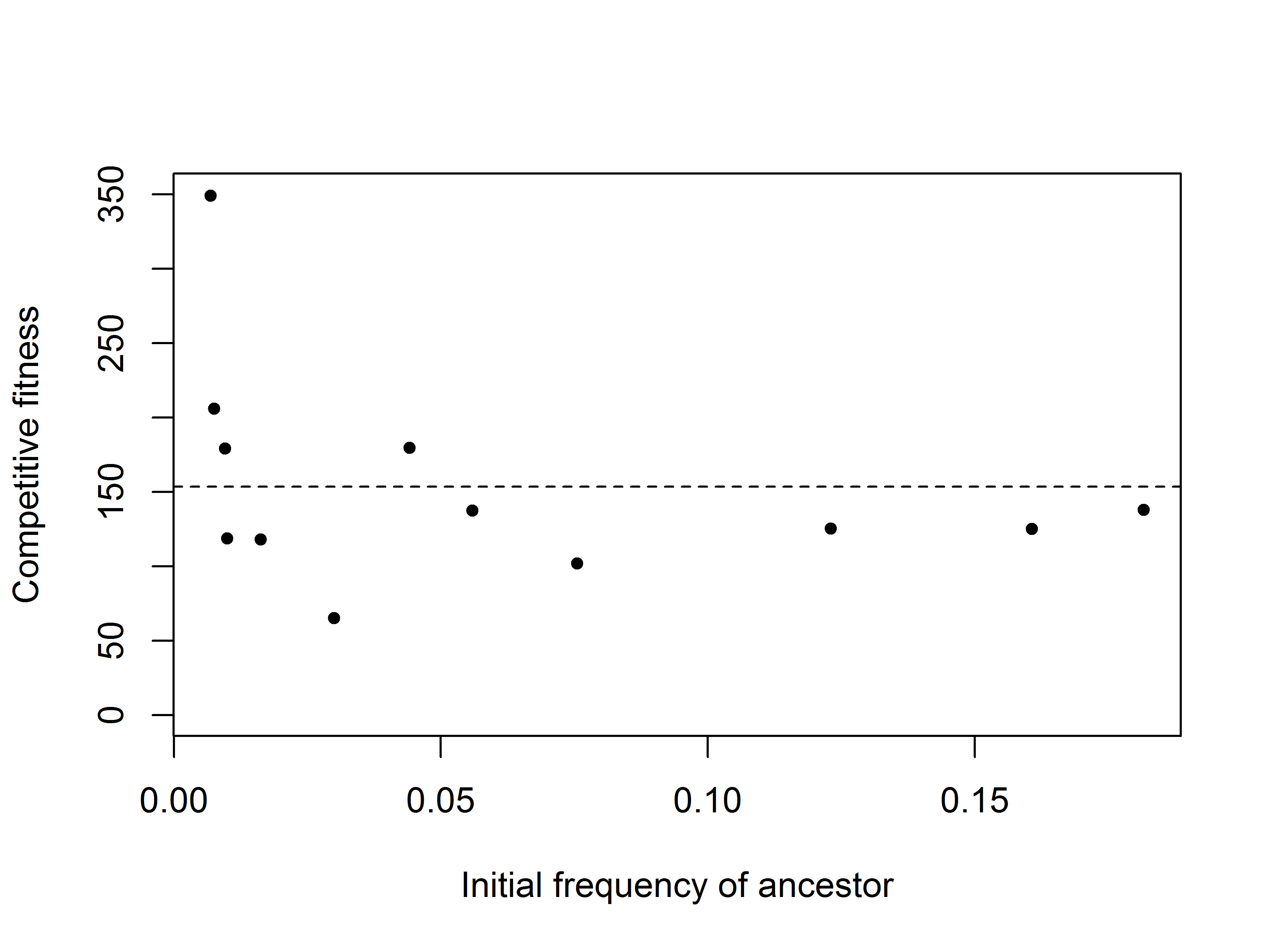

Supplement: S3 Fig — Competitions were initialized with target starting ratios of ancestor: common competitor at 1: 5, 1: 10, 1: 50, and 1: 100. (An initial ratio of 1: 1 yielded a final concentration of common competitor that was too low for reliable fitness estimates.) Competitive fitness was calculated using Eq 2. The mean competitive fitness is indicated with a dashed line. The competitive fitness values of the ancestral genotype are not significantly correlated with its initial frequency (Pearson’s correlation test, ρ = -0.32, p = 0.30). (TIF) [file pone.0189602.s007.tif]

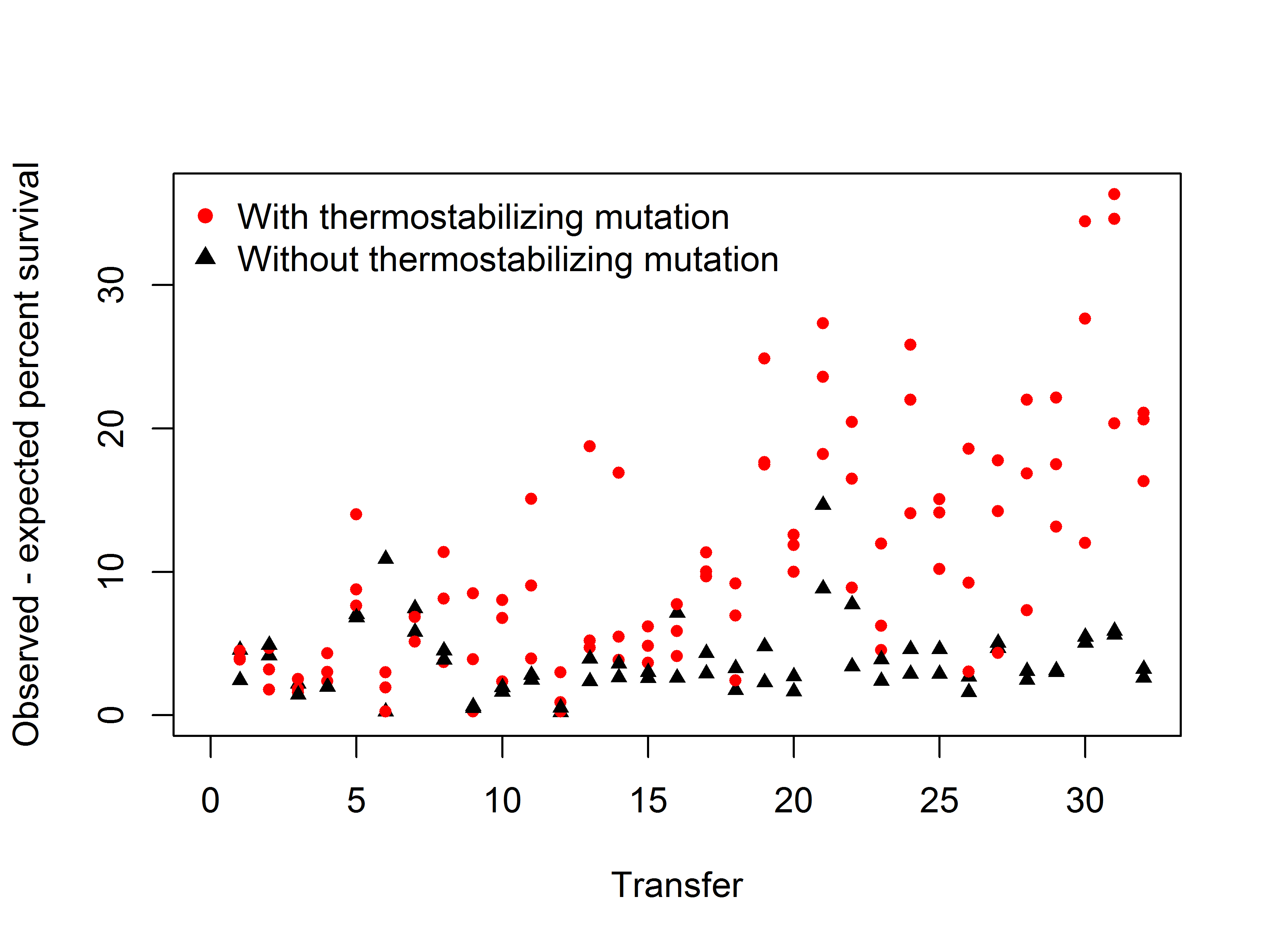

Supplement: S4 Fig — At each transfer, the survival of each population (Observed percent survival) was compared to the percent survival of the ancestor (Expected percent survival) at 50°C. Lineages are distinguished according to whether at least one thermostabilizing mutation was present in the endpoint population. (Note that, although the entire lineage has been colored for the purpose of visualization, the exact point in time at which the thermostabilizing mutation arose was not evaluated in this study.) (TIF) [file pone.0189602.s008.tif]
