# Supplementary material for: Adaptations of an RNA virus to increasing thermal stress
Source: PLoS One. 2017 Dec 21;12(12):e0189602. doi: 10.1371/journal.pone.0189602 (PMC5739421; doi:10.1371/journal.pone.0189602)
Supplement: S1 Text — (PDF) [file pone.0189602.s009.pdf]

S1 Text. Effects of additional mutations in the engineered viral mutants on thermostability and relative competitive fitness.

Because Φ6 mutates rapidly, several engineered genotypes used in this study contained additional mutations in regions other than the locus of interest. Often, these additional mutations were in extended *t*-runs in the 3' untranslated region (UTR) of the S segment and were most likely due to slippage during viral replication. (The specific mutations are recorded in S1 Table.) Because these slippage mutations occurred commonly, we were sometimes able to isolate pairs of mutants containing the same focal mutation, one of which had specific 3' UTR mutations and the other of which did not.

To account conservatively for the effects of the 3' UTR mutations on thermostability (Fig A-C), we exposed cell-free lysates to a 5-minute heat shock at temperatures ranging from 25°C-55°C and plated before and afterward to calculate percent survival. We then fit Equation 1 to the data, estimating the parameters T_{50} (intersection of curve with dotted line) and n by maximum likelihood. For each pair, a first model was fit to the combined data (mutant without 3' UTR mutations + mutant with 3' UTR mutations; black). A second model then estimated a separate T_{50} and n for each lysate (blue, mutant without 3' UTR mutations; red, mutant with 3' UTR mutations).

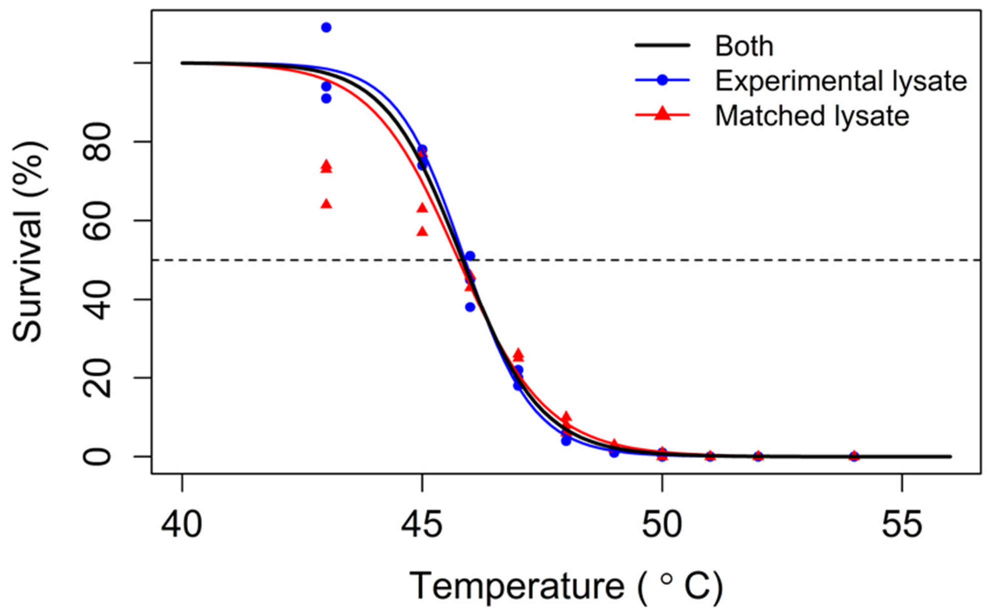

Fig A. Thermal kill curve comparisons between the isolated ancestral genotype and an independently engineered plaque with identical sequence, to account for plaque-specific effects.

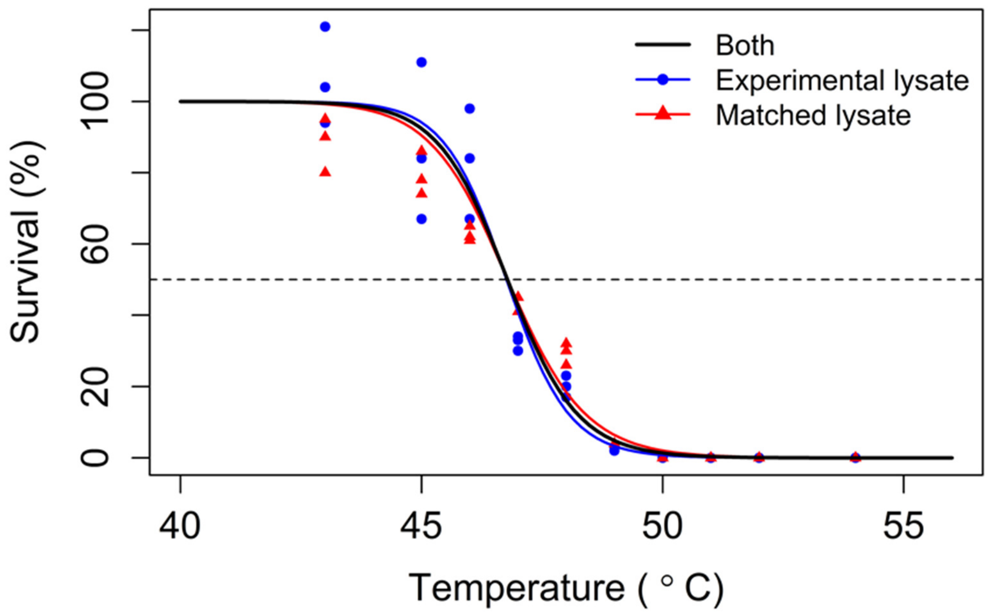

Fig B. Thermal kill curve comparison of two isolates with P8 V109I, one of which also contained a double-*t* deletion at position 2443 in the 3' UTR.

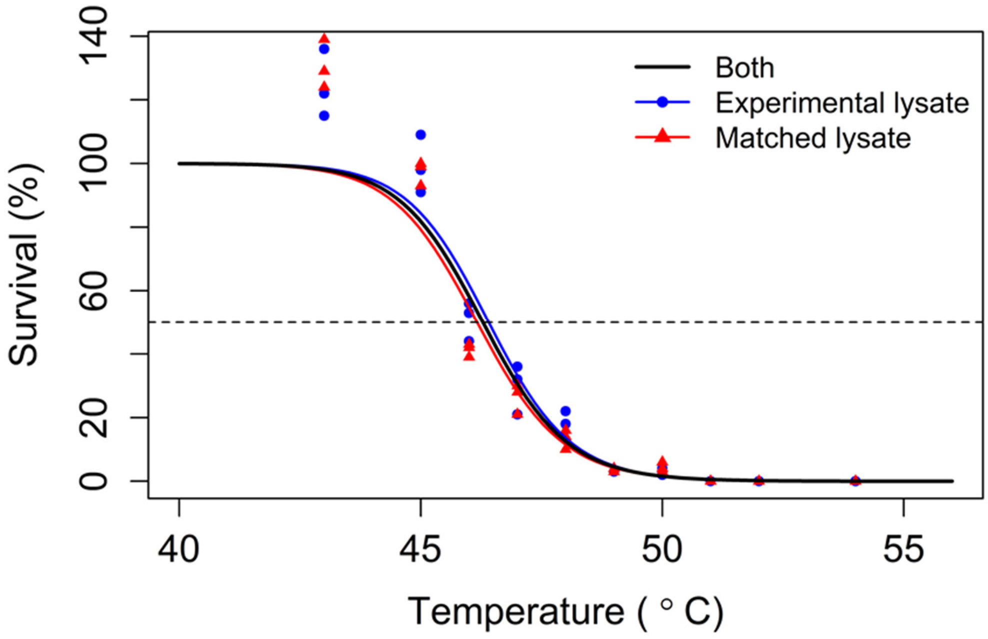

Fig C. Thermal kill curve comparison of two isolates with P5 R124S + P5 E201K, one of which also contained a *t* insertion at position 2376 and a *t* deletion at position 2443 in the 3' UTR.

In two cases (Figs A-B), the latter model was a better fit to the data after Bonferroni correction, suggesting that the additional mutation affected viral thermostability. However, subsequent tests that allowed either T_{50} or n to vary while holding the other parameter constant indicated that the T_{50} estimate was not significantly different in lysates with the 3' UTR mutations (see the Figshare Data Repository). (We note that the T_{50} values of the experimental mutants are highly correlated regardless of whether our model allows only T_{50} to vary or both T_{50} and n, so this choice of model does not alter any of the conclusions of our study. See also Data Repository.)

We also competed the mutants with the 3' UTR mutations against a common competitor to evaluate their relative competitive fitness in comparison to the ancestral genotype (Fig D). In these cases, relative competitive fitness was compared to the genotype with the same focal

mutation but lacking the 3' UTR mutations. We found no significant effect of the 3' UTR mutations on the relative competitive fitness of the viruses.

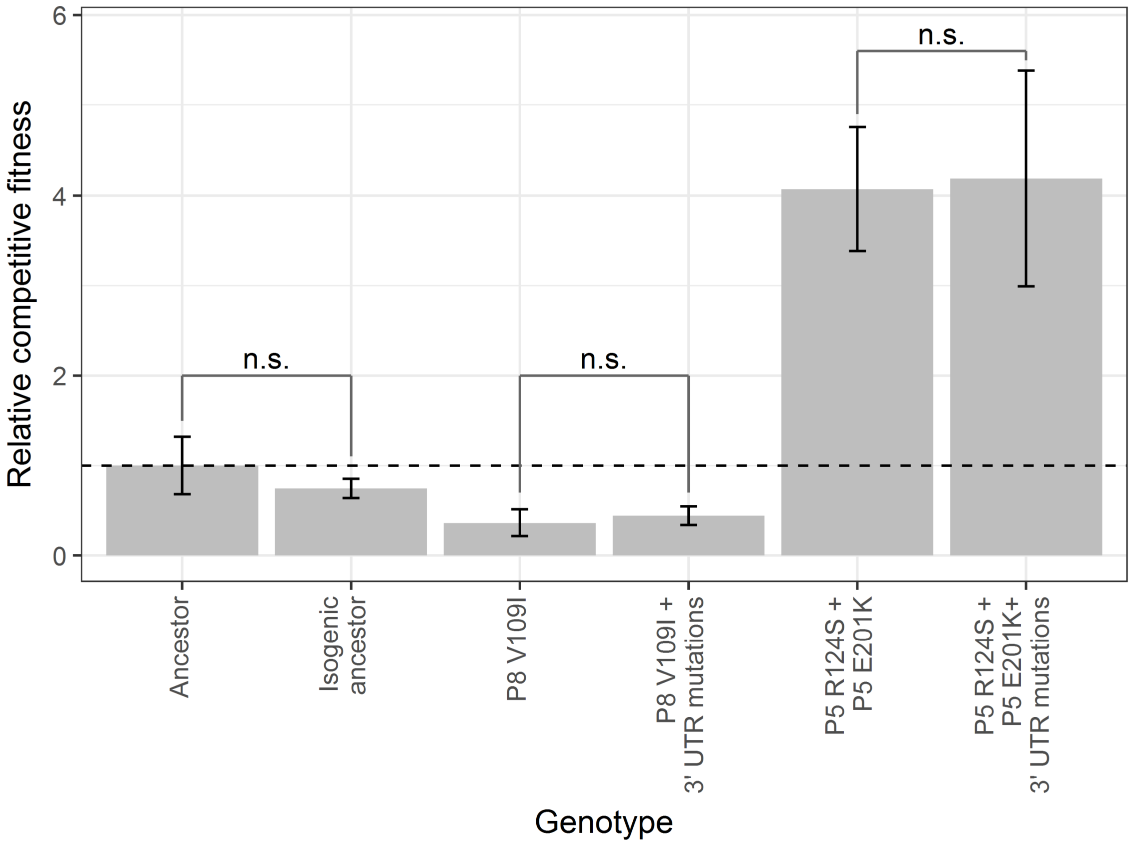

Fig D. Competitive fitness of all sets of matched mutants, relative to the ancestral genotype. Bar heights indicate the mean of three replicate competitions; error bars denote standard deviation. Pairs that share focal amino acid mutation(s) do not differ significantly in fitness (2-sample t-test, $p > 0.3$ for all pairs).
