# Supplementary material for: Adaptations of an RNA virus to increasing thermal stress
Source: PLoS One. 2017 Dec 21;12(12):e0189602. doi: 10.1371/journal.pone.0189602 (PMC5739421; doi:10.1371/journal.pone.0189602)
Supplement: S2 Text — (PDF) [file pone.0189602.s010.pdf]

S2 Text. Historical sequencing of population Gradual 1

We sequenced lineage G1 (Replicate population 1 from the Gradual treatment) back in time to understand when its mutations arose (Table A).

Table A. Sanger sequencing results of mutations in P5 and P8 in the G1 lineage over time.

Transfer	Mutations detectable
8	None
16	b / B<br>c / C1
24	C1
28	b / B<br>C1 / C2
30	a / A<br>b = B<br>C1 / C2
31	a / A<br>b = B<br>c = C1 = C2
32	a = A<br>b = B<br>c = C2

The ancestral genotype is denoted with lower case (a, b, c), while mutations are denoted with upper case (mutation A = P8 Q69R, B = P5 R124S, C1 = P5 E201Q, C2= P5 E201K). Polymorphisms (a double peak in the chromatogram) are denoted by listing both alleles. A slash separating the alleles indicates that the peak for the first allele was higher than the peak for the second allele, while an equals sign indicates that the peak heights were roughly equivalent.

From these data, we concluded that the R124S mutation in P5 arose relatively early in evolution (prior to the halfway point, Transfer 16). An additional mutation (not evaluated in this study), E201Q in P5, arose separately between Transfers 8 and 16. The genotype with this mutation increased in frequency through Transfer 24. Its frequency then declined as the mutations P8 Q69R and P5 E201K, presumably on a genetic background with P5 R124S, rose to appreciable frequencies in the final 4-6 days of evolution.
